# A126 in the active site and TI167/168 in the TI loop are essential determinants of the substrate specificity of PTEN

**DOI:** 10.1007/s00018-018-2867-z

**Published:** 2018-07-09

**Authors:** Michael G. Leitner, Kirstin Hobiger, Angeliki Mavrantoni, Anja Feuer, Johannes Oberwinkler, Dominik Oliver, Christian R. Halaszovich

**Affiliations:** 1 Institute of Physiology and Pathophysiology, Philipps-University Marburg, Deutschhausstr. 1-2, 35037 Marburg, Germany; 2 Division of Physiology, Department of Physiology and Medical Physics, Medical University of Innsbruck, 6020 Innsbruck, Austria; 3 DFG Research Training Group GRK 2213, Membrane Plasticity in Tissue Development and Remodeling, Philipps-University Marburg, 35043 Marburg, Germany; 4 Center for Mind, Brain and Behavior (CMBB), Universities of Marburg and Giessen, Marburg/Giessen, Germany

**Keywords:** Voltage-sensitive phosphatases, Protein tyrosine phosphatases (PTPs), Ci-VSP, Hs-VSP, Neomorphic mutations, Phosphoinositides, Phosphoinositide signaling

## Abstract

PTEN prevents tumor genesis by antagonizing the PI3 kinase/Akt pathway through D3 site phosphatase activity toward PI(3,4)P_2_ and PI(3,4,5)P_3_. The structural determinants of this important specificity remain unknown. Interestingly, PTEN shares remarkable homology to voltage-sensitive phosphatases (VSPs) that dephosphorylate D5 and D3 sites of PI(4,5)P_2_, PI(3,4)P_2_, and PI(3,4,5)P_3_. Since the catalytic center of PTEN and VSPs differ markedly only in TI/gating loop and active site motif, we wondered whether these differences explained the variation of their substrate specificity. Therefore, we introduced mutations into PTEN to mimic corresponding sequences of VSPs and studied phosphatase activity in living cells utilizing engineered, voltage switchable PTEN_CiV_, a Ci-VSP/PTEN chimera that retains D3 site activity of the native enzyme. Substrate specificity of this enzyme was analyzed with whole-cell patch clamp in combination with total internal reflection fluorescence microscopy and genetically encoded phosphoinositide sensors. In PTEN_CiV_, mutating TI167/168 in the TI loop into the corresponding ET pair of VSPs induced VSP-like D5 phosphatase activity toward PI(3,4,5)P_3_, but not toward PI(4,5)P_2_. Combining TI/ET mutations with an A126G exchange in the active site removed major sequence variations between PTEN and VSPs and resulted in D5 activity toward PI(4,5)P_2_ and PI(3,4,5)P_3_ of PTEN_CiV_. This PTEN mutant thus fully reproduced the substrate specificity of native VSPs. Importantly, the same combination of mutations also induced D5 activity toward PI(3,4,5)P_3_ in native PTEN demonstrating that the same residues determine the substrate specificity of the tumor suppressor in living cells. Reciprocal mutations in VSPs did not alter their substrate specificity, but reduced phosphatase activity. In summary, A126 in the active site and TI167/168 in the TI loop are essential determinants of PTEN’s substrate specificity, whereas additional features might contribute to the enzymatic activity of VSPs.

**Electronic supplementary material:**

The online version of this article (10.1007/s00018-018-2867-z) contains supplementary material, which is available to authorized users.

## Introduction

PTEN (phosphatase and tensin homologue deleted on chromosome 10) is a well-characterized D3-phosphoinositide (PI) phosphatase that dephosphorylates PI(3,4)P_2_ and PI(3,4,5)P_3_ to PI(4)P and PI(4,5)P_2_, respectively [1–6]. In doing so, PTEN prevents tumorigenesis by antagonizing growth factor-stimulated phosphoinositide 3-kinases (PI3K) [2, 7]. Importance of PTEN is highlighted by the fact that various PTEN mutations were found in numerous tumors [6, 8, 9], but also in epilepsy [10], autism spectrum disorders [11–15], and Alzheimer’s disease [16, 17]. Despite physiological relevance, little is known about the structural origin for this catalytic specificity.

**Fig. 1:**
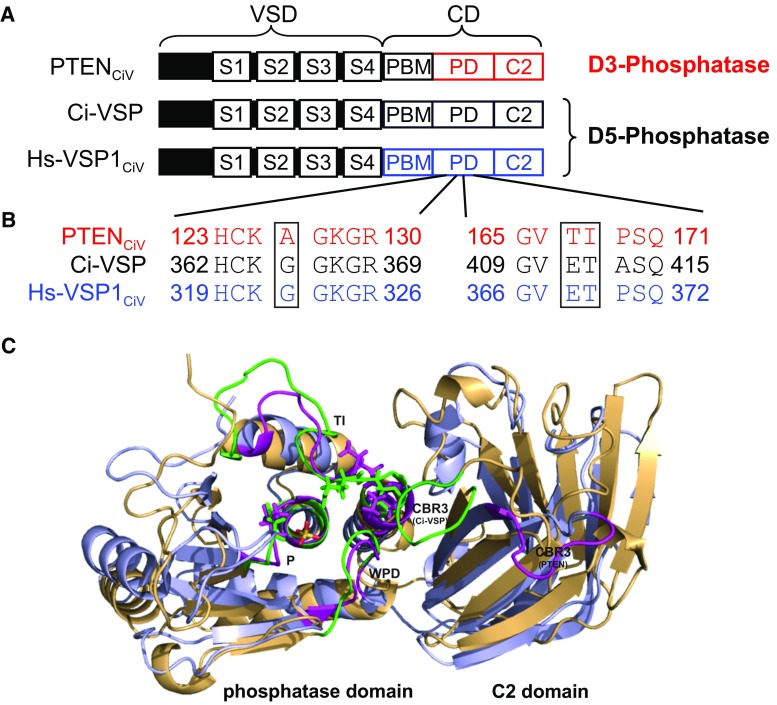
Schematic representation and structural alignment of engineered voltage-sensitive phosphatases. **a** Schematic representation of the VSPs used in this study. The chimeras consist of the N-terminal voltage-sensor domain (VSD) of Ci-VSP and the C-terminal catalytic domain (CD) of PTEN or Hs-VSP1 (S1–S4, transmembrane domains; PBM, phospholipid-binding motif; PD, phosphatase domain; C2, C2 domain). **b** Sequence alignment of P loop (HCX_5_R- or active site motif) and TI loop (PTEN)/gating loop (Ci-VSP and Hs-VSP1_CiV_). Note that sequence differences between the D3-site phosphatase PTEN_CiV_ and the D5-site phosphatases Ci-VSP and Hs-VSP1_CiV_ are restricted to A126 in PTEN corresponding to G365 in Ci-VSP and G322 in Hs-VSP1, and to TI167/168 in PTEN that is ET411/412 in Ci-VSP and ET368/369 in Hs-VSP1. **c** Structural alignment of PTEN (in blue; PDB 1d5r [32]) and the catalytic domain of Ci-VSP (in orange; homology model based on molecular dynamics simulations as described earlier [36]) with a phosphate ion bound to the active site. Flexible motifs shaping the substrate binding pocket (P, TI and WPD loop) and the CBR3 loop of the C2 domain are highlighted in green and magenta for Ci-VSP and PTEN, respectively. The A/G position in the P loop and the TI/ET pair in the TI/gating loop are depicted as sticks

Interestingly, PTEN shows high sequence similarity to the catalytic domain (CD) of voltage-sensitive phosphatases (VSPs; Fig. S1), such as Ci-VSP [18], Dr-VSP [19], Xl-VSP1 and Xl-VSP2 [20], and the human orthologue Hs-VSP1 (previously named hVSP1, TPTE2 or TPIP) [21–23]. VSPs are controlled by membrane voltage, such that depolarization increases phosphatase activity. The enzymatic activity of Ci-VSP [24, 25] and Hs-VSP1 [23, 26] has been characterized in living cells, identifying them as PI(4,5)P_2_ and PI(3,4,5)P_3_ D5-phosphatases. Data on Xl-VSP1 and Xl-VSP2 [20] and Dr-VSP [19] gave further evidence for D5-phosphatase activity toward PI(4,5)P_2_ of VSPs in general. However, recent data expanded the initial concept of VSPs being pure D5 site-specific phosphatases, since D3 activity toward PI(3,4)P_2_ and PI(3,4,5)P_3_ was revealed for Ci-VSP [26–30]. These findings indicate less specific activity of VSPs compared to the highly specific D3 phosphatase PTEN. Given high sequential and structural homology of VSPs and PTEN (Fig. 1), the variation in their substrate specificity is surprising and raises the question of the structural origin of this difference.

PTEN and VSPs belong to the protein tyrosine phosphatase (PTP) superfamily due to the HCX_5_R motif in their active site (called “PTP recognition” or “P loop”). These enzymes share canonical structural properties in the phosphatase domain, with three loops forming the substrate binding pocket (Fig. 1) [8, 31]. Structural data available for PTEN [32, 33] and Ci-VSP [27, 34] indicated that P loop and two other motifs (the TI/gating and the WPD loop), which surround the active site (Fig. 1c), dynamically shape the size and depth of the substrate binding pocket. Therefore, residues within these loops might play a crucial role in determining the substrate specificity of these phosphatases. Indeed, amino acids lining the active site pocket of PTEN differ significantly only in two places from those found in VSPs: (i) in the HCX_5_R motif of the P loop, and (ii) in the TI/gating loop (Fig. 1b, Fig. S1).

(i) The P loop sequences of PTEN and VSPs vary in only one position. Where PTEN contains an alanine (A126), VSPs carry a glycine at the homologous position (Fig. 1b). Recently, we found that the A126G mutation shifted the specificity of PTEN from D3 to D5 site activity toward PI(3,4,5)P_3_ [35]. Interestingly, this gain-of-function mutation stimulated the PI3K/Akt proliferation pathway and potential tumor growth in a prostate cancer patient [35]. As PTEN(A126G) did not recognize PI(4,5)P_2_ as substrate, the single amino acid exchange in the P loop is sufficient to alter site specificity, but not substrate specificity of PTEN. It has already been speculated that the homologous exchange of glycine to alanine (G/A mutations) in Ci-VSP converts its pre-dominant D5 activity into activity towards the D3 site [24]. However, respective results on VSP mutants obtained in different cell types and in vitro are inconsistent [23, 27] and thus need further clarification.
(ii) The so-called “TI loop” (named after the threonine/isoleucine pair in PTEN; also called “gating loop” in VSPs) differs in length and sequence between PTEN and VSPs (Fig. S1). In particular, the TI pair in PTEN is replaced by glutamic acid/threonine (ET) in the corresponding loop of VSPs. The TI/gating loop was already proposed to interfere with enzymatic activity in both phosphatases [27, 32, 34, 36]. Based on crystallography data of the soluble catalytic domain of Ci-VSP, it was suggested that the glutamate at position 411 regulates access for the substrate to the active site and, therefore, might interfere with substrate specificity [27, 34]. Importantly, however, the molecular mechanism behind this regulation is not fully understood.

Since available structural data do not provide a satisfying explanation for the relevance of residues in the P and TI loop, we aimed at clarifying their role for determining the substrate specificity of the phosphatases. For this, we employed well-characterized Ci-VSP and engineered chimeric voltage-sensitive enzymes PTEN_CiV_ (originally named “Ci-VSPTEN16”) [37] and Hs-VSP1_CiV_ [23]. In these chimeric proteins, the CD of Ci-VSP was replaced by that of PTEN or Hs-VSP1 enabling direct control of phosphatase activity by membrane voltage while retaining the substrate specificity of the native enzymes [23, 37].

We performed systematic step-by-step exchanges of amino acids in the P and the TI/gating loop of PTEN_CiV_ into corresponding residues of VSPs and vice versa. Substrate specificity and enzymatic activity of the phosphatases were measured using the whole-cell patch clamp technique combined with total internal reflection fluorescence (TIRF) microscopy in living cells. We found that alanine at position 126 in the P loop and the TI pair in the TI loop independently determine substrate specificity of PTEN_CiV_: exchanging these amino acids individually to corresponding residues of VSPs conferred VSP-like D5 activity toward PI(3,4,5)P_3_ to otherwise D3 site-specific PTEN_CiV_, although the phosphatase remained inactive toward PI(4,5)P_2_. Strikingly, the simultaneous exchange in both loops, which removed major sequence differences between VSPs and PTEN, completely converted PTEN into a D5 phosphatase with the apparent substrate specificity of native VSPs. In contrast, reciprocal mutations in VSPs reduced or even abolished phosphatase activity indicating the existence of a distinct mechanism for substrate specificity in these enzymes.

## Materials and methods

### Molecular biology

The construction of chimeras containing the VSD of Ci-VSP and the cytosolic domains of either PTEN or Hs-VSP1 was described elsewhere [23, 37]. Hs-VSP1_CiV_(D136N) was used in all experiments (termed here as “Hs-VSP1_CiV_”), since the voltage dependence of this mutant is comparable to that of Ci-VSP [23]. Point mutations were introduced by site-directed mutagenesis. All cDNA sequences were verified before use (Seqlab Laboratories, Göttingen, Germany). Throughout the manuscript, the term “wild type” denotes absence of mutations in the catalytic domain, also with respect to the chimeric enzymes.

### Cell culture and expression of proteins of interest

Chinese hamster ovary (CHO) cells were grown as described before [25], plated onto glass bottom dishes (WillCo Wells B. V., Amsterdam, The Netherlands) or cover slips, and transfected using jetPEI (Polyplus Transfection, Illkirch, France). Madin–Darby Canine Kidney (MDCK) cells were grown in DMEM GlutaMax (Gibco/ThermoFisher Scientific, Darmstadt, Germany), supplemented with 10% (vol/vol) FBS and 1% (vol/vol) Pen/Strep. MDCK cells were seeded on cover slips in 35 mm diameter cell culture dishes and transfected using Lipofectamine 2000 (ThermoFisher Scientific). PC-3 cells were grown as described before [35], seeded in 35–60 mm diameter cell culture dishes and transfected using jetPET (Polyplus Transfection). Expression vectors used for transfection were: PLCδ_1_-PH (UniProt accession number P51178), and Btk-PH (Q06187) in pEGFP-N1 vector; TAPP1-PH (Q9HB21) in FUGW vector (contains eGFP); bovine phosphatidylinositol 3-kinase p110α (constitutively active mutant K227E; P32871) [38]; Ci-VSP (Q4W8A1) or chimeras in pmRFP-C1 vector; KCNQ2 (K_v_7.2) (O43526) in pBK-CMV. For confocal imaging and PI(3,4)P_2_ mass ELISA, non-chimeric human PTEN wild type (P60484) or mutated PTEN were cloned in the pmRFP-C1 vector. All experiments were performed 24–48 h after transfection.

### Electrophysiology

In TIRF experiments, the whole-cell configuration of the patch clamp technique was employed using an EPC-10 amplifier (HEKA Elektronik, Lambrecht Germany) controlled by PatchMaster software (HEKA) on a Mac mini (Apple Inc, Cupertino, CA, USA). Cells were clamped to − 60 mV and phosphatases were activated by depolarizing the membrane potential to + 80 mV. In these experiments, series resistance (*R*_s_) typically was below 5 MΩ and no *R*_s_ compensation was applied.

KCNQ2 (K_v_7.2) currents were recorded in the whole-cell configuration with an AxoPatch 200B amplifier (Molecular Devices, Sunnyvale, CA, USA) in conjunction with an ITC-18 interface (HEKA) controlled by PatchMaster software on a PC running Microsoft Windows. Currents were sampled at 5 kHz and low-pass filtered at 2 kHz. *R*_s_ was typically below 6 MΩ and was compensated throughout the recordings (80–90%). KCNQ2 currents were elicited by a voltage step from the holding potential of − 60 to 0 mV every 5 s and phosphatases were activated at + 80 mV in between these steps. Data were analyzed using PatchMaster (HEKA) and IgorPro (Wavemetrics, Lake Oswego, OR, USA).

Patch pipettes were pulled from borosilicate glass using a P2000 puller (Sutter Instrument Company, Novato, CA, USA) and had an open pipette resistance between 2 and 4 MΩ after back-filling with intracellular solution containing (in mM) 135 KCl, 2.41 CaCl_2_ (100 nM free Ca^2+^), 3.5 MgCl_2_, 5 EGTA, 5 HEPES and 2.5 Na_2_-ATP, pH 7.3 (with KOH), 290–295 mOsm/kg. Throughout the recordings, cells were kept in extracellular solution containing (in mM) 144 NaCl, 5.8 KCl, 0.9 MgCl_2_, 1.3 CaCl_2_, 0.7 NaH_2_PO_4_, 5.6 d-glucose, and 10 HEPES, pH 7.4 (with NaOH), 305–310 mOsm/kg. All experiments were performed at room temperature (22–25 °C).

### TIRF imaging

The majority of TIRF experiments were performed using an upright TIRF setup, as described before [25]. Briefly, a BX51WI upright microscope (Olympus) equipped with a TIRF condenser (NA 1.45; Olympus) and a 488 nm laser (20 mW; Picarro, Sunnyvale, CA, USA) was used. Fluorescence was imaged through a LUMPlanFI/IR 40×/0.8 NA water immersion objective. Images were acquired with a TILL-Imago QE cooled CCD camera (TILL photonics, Gräfelfing, Germany) in combination with a Polychrom IV light source (TILL photonics) controlled by TILLvision software (TILL photonics). The laser shutter was controlled by the Polychrom IV.

**Fig. 2:**
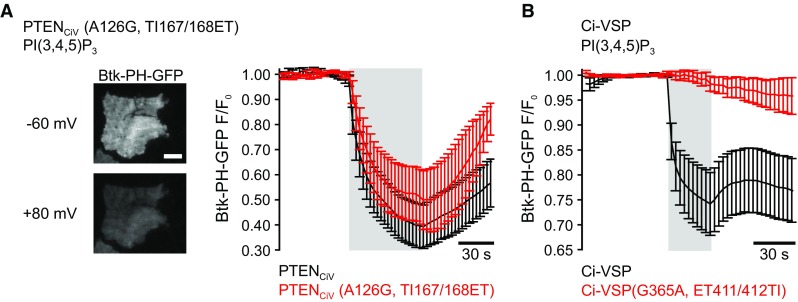
PTEN_CiV_(A126G,TI167/168ET) is a PI(3,4,5)P_3_-phosphatase, whereas Ci-VSP(G365,ET411/412TI) lacks detectable PI(3,4,5)P_3_-phosphatase activity. **a** Left panel, representative TIRF image of a CHO cell co-expressing mRFP-PTEN_CiV_(A126G, TI167/168ET) and Btk-PH-GFP before (top) and during (bottom) depolarization of the cell membrane through whole-cell patch clamp (scale bar represents 10 μm). Right panel, summarized TIRF signals in response to step depolarization of the holding potential from − 60 to + 80 mV (gray shading) of cells co-expressing wild type mRFP-PTEN_CiV_ (black trace, *n* = 6) or mRFP-PTEN_CiV_(A126G, TI167/168ET) (red, *n* = 5) together with the PI(3,4,5)P_3_ sensor Btk-PH-GFP. **b** Summarized TIRF signals from cells co-expressing wild type mRFP-Ci-VSP (black, *n* = 9) or mRFP-Ci-VSP(G365A, ET411/412TI) (red, *n* = 7) together with the PI(3,4,5)P_3_ sensor Btk-PH-GFP

For experiments shown in Fig. 2a, an inverted TIRF setup was used. A Nikon Eclipse Ti inverted microscope was equipped with an Apo TIRF 100×/1.49 oil immersion objective, a TIRF condenser (Rapp OptoElectronic, Wedel, Germany) and a 488 nm laser (200 mW, iBeam smart; Toptica Photonics AG, Gräfelfing, Germany). Images were acquired with a CoolSnap HQ2 camera (Photometrics, Tuscon, AZ, USA). The laser shutter (nmLaser Products Inc., San José, CA, USA) was controlled by the camera. Image acquisition was performed using the µ-Manager software [39].

For all TIRF experiments, the frame interval was 3 s and the electrophysiology setup was synchronized to the imaging setup. Cells were co-transfected with plasmids encoding a mRFP-tagged phosphatase, a PI sensor domain, and—in case of the PI(3,4)P_2_ or PI(3,4,5)P_3_ binding domains—additionally with constitutively active PI3-kinase p110α(K227E) [38]. Experiments were done 24–48 h post-transfection on cells selected for co-expression of the mRFP-tagged phosphatase and the GFP-tagged PI sensor probe. Cells under investigation were whole-cell patch clamped as described above. Experiments were carried out at room temperature (22–25 °C). Imaging data were analyzed using TILLvision (Till photonics), ImageJ/Fiji (NIH, Bethesda) [40, 41], and IgorPro (Wavemetrics). Regions of interest (ROIs) encompassed the footprint of a single cell excluding cell margins to avoid movement artifacts. *F*/*F*_0_ traces were calculated from the background-corrected TIRF signal intensity *F*, normalized to the initial intensity *F*_0_, which was calculated as the average over the baseline interval, by averaging over the ROI. *F*/*F*_0_ traces were corrected for bleaching according to mono-exponential fits to the baseline interval as described previously [37].

### Confocal microscopy

Confocal imaging was performed with an upright LSM 710—Axio Examiner.Z1 microscope equipped with a W-Plan-Apochromat 63×/1.0 M27 water immersion objective (Carl Zeiss, Jena, Germany) as described before [42]. Red fluorescent protein (RFP) was excited at 561 nm with a DPSS 561-10 laser (Zeiss) and fluorescence emission was detected at 582–754 nm. Green fluorescent protein (GFP) was excited at 488 nm with an argon laser and fluorescence emission was detected at 493–556 nm. For confocal imaging, RFP-tagged PTEN and TAPP1-PH-GFP were co-expressed in MDCK cells. Confocal images were processed and montages were generated using ImageJ [40]. Subcellular localization of TAPP1-PH-GFP in MDCK cells co-expressing PTEN wild type, PTEN(C124S) or PTEN(A126G, TI167/168ET) was analyzed by blinded counting of cells with membrane association of TAPP1-PH-GFP.

### Quantification of PI(3,4)P_2_ levels

PI(3,4)P_2_ was quantified in lysates of PC-3 cells expressing RFP-tagged PTEN wild type, catalytic dead (C124S) or triple mutant (A126G, TI167/168ET) using the PI(3,4)P_2_-mass ELISA kit #K-3800 by Echelon Biosciences (U.S.). 24 h before transfection, 1 × 10^6^ cells were seeded in 60-mm dishes (in total 5 dishes per construct). Per dish, cells were transfected with 5 µg DNA pre-mixed with 10 µL jetPEI (Polyplus Transfection, France) in 500 µL 150 mM NaCl. 24 h after transfection, cells were trypsinized, pooled, and washed once in 5 mL PBS/10% (vol/vol) FBS (centrifugation: 2 min, 1200×*g*, 4 °C). Cells then were resuspended in 1 mL PBS/10% (vol/vol) FBS and sorted for RFP-PTEN expression using a BD FACSAria II system (BD Biosciences, San Jose, USA) with an excitation wavelength at 561 nm. After sorting, RFP positive cells were centrifuged for 2 min, 1200×*g* at 4 °C, resuspended in 3 mL cell culture media, and seeded in 35-mm dishes. Cells were harvested 24 h later by trypsinization and washed once in 1 mL PBS/10% (vol/vol) FBS (1200×*g*, 2 min, 4 °C). Before lipid extraction, vital cells were counted (using trypan blue), and per batch, cell numbers of all samples were adjusted to the same level by dilution in 0.5–1 mL PBS. Final cell numbers between different batches varied from 1 to 4 × 10^4^. 1.5–3 mL ice cold 0.5 M TCA was added to cell suspensions, and after 5 min of incubation on ice, cells were centrifuged at 1000×*g* for 7 min at room temperature (RT). Pellets were resuspended in 2 mL 5% (vol/vol) TCA/1 mM EDTA, vortexed for 2 min, and centrifuged at 1000×*g*, 5 min at RT. This step was repeated once and supernatant was discarded. Neutral lipids were extracted by resuspending the pellet in 1 mL MeOH:CHCl_3_ (2:1), vortexing for 3 × 10 min (with short spin-down between the vortex steps), and final centrifugation at 847×*g* for 5 min at RT. This step was performed twice. Afterwards, acidic lipids were extracted by adding 150 µL MeOH:CHCl_3_:12 M HCl (80:40:1), vortexing for 4 × 15 min (with short spin-down between the vortex steps), and final centrifugation at 847×*g*, 5 min at RT. Supernatants were transferred into new 2-mL vials. For phase separation, 450 µL of 0.1 M HCl and 250 µL CHCl_3_ were added. Samples were vortexed for 30 s and centrifuged for 5 min with 847xg at RT. The organic (lower) phases were collected into 2-mL glass vials and dried under vacuum in a desiccator overnight at RT. Dried lipid films were stored at − 20 °C until usage. Lipids were thawed at RT and then dissolved by adding 0.5–1 mL pre-warmed (50 °C) PBS-Tween + 3% protein stabilizer (provided by the Echelon kit), vortexing for 5 min at RT, and 5 freeze/thaw cycles (freezing in liquid nitrogen, 10 min at − 20 °C, thawing at 50 °C for 5 min in a water bath, and vortexing for 5 min at RT). Before adding to a 96-well ELISA incubation plate, samples were spun down. PI(3,4)P_2_-quantification assay was performed in accordance to the manufacturer’s instruction protocol by measuring samples in triplets.

### Data analysis/statistics

Data are presented as mean ± SEM with n indicating the number of independent recordings (individual cells). Statistical analysis of electrophysiological data was performed with *t* test or Scheffé/Dunnett test for multiple comparisons. Analysis of PI(3,4)P_2_-quantification was done with two-sample *t* tests without correction for multiple comparisons due to the low numbers of comparisons and to minimize the risk for unwanted type-II errors [43]. Statistical significance was assigned at *P* ≤ 0.05 (*P ≤ 0.05, **P ≤ 0.01, ***P ≤ 0.001).

## Results

Following established techniques to assess the substrate specificity of voltage-sensitive PI phosphatases in living cells [23, 25, 37], we expressed the enzymes (chimeric PTEN_CiV_ and Hs-VSP1_CiV_, Ci-VSP) together with GFP-tagged PI-binding domains in CHO cells. Under whole-cell voltage clamp to control the activity of the phosphatases, translocation of fluorescent PI sensor domains between plasma membrane and cytosol was monitored using TIRF microscopy. Since this technique allows for selective excitation of fluorophores at the cell membrane, the observed fluorescence intensity reflects the amount of membrane-bound PI sensor molecules, which directly correlates with the respective PI concentration at the membrane surface (Fig. 2a). We utilized the Btk-PH domain for detection of PI(3,4,5)P_3_, TAPP1-PH for PI(3,4)P_2_, and PLCδ_1_-PH for PI(4,5)P_2_ [44–47]. Additionally, activity of PI(4,5)P_2_-sensitive voltage-gated KCNQ2 (K_v_7.2) K^+^ channels was taken as an independent measure for the PI(4,5)P_2_ concentration in the plasma membrane [48].

### PTEN_CiV_(A126G, TI167/168ET), but not reciprocal VSP mutants, exhibit activity towards PI(3,4,5)P_3_

Activation of wild type PTEN_CiV_ and Ci-VSP by depolarization of the membrane potential led to dissociation of the PI(3,4,5)P_3_-specific probe Btk-PH-GFP from the plasma membrane, as shown by a decrease of the TIRF signal (Fig. 2). These results demonstrated PI(3,4,5)P_3_ depletion through PTEN_CiV_ and Ci-VSP, as previously reported [25, 37].

In PTEN_CiV_, simultaneous insertion of the A126G and the TI167/168ET exchange in P and TI loop, respectively, thereby matching the sequence of VSPs with regard to amino acid residues lining the active site, preserved activity towards PI(3,4,5)P_3_ (Fig. 2a). In contrast, reciprocal mutations in Ci-VSP abolished phosphatase PI(3,4,5)P_3_ activity (Fig. 2b).

These findings demonstrated that PTEN(A126G, TI167/168ET) exhibited activity toward PI(3,4,5)P_3_. However, these experiments did not give any insight into site-specific activity of the PTEN_CiV_ mutants. Thus, we subsequently used reporters specific for PI(3,4)P_2_ or PI(4,5)P_2_ to clarify this question.

### PTEN_CiV_ (A126G, TI167/168ET), PTEN_CiV_(TI167/168ET) and PTEN_CiV_(A126G) are D5 phosphatases in contrast to wild type PTEN_CiV_

**Fig. 3:**
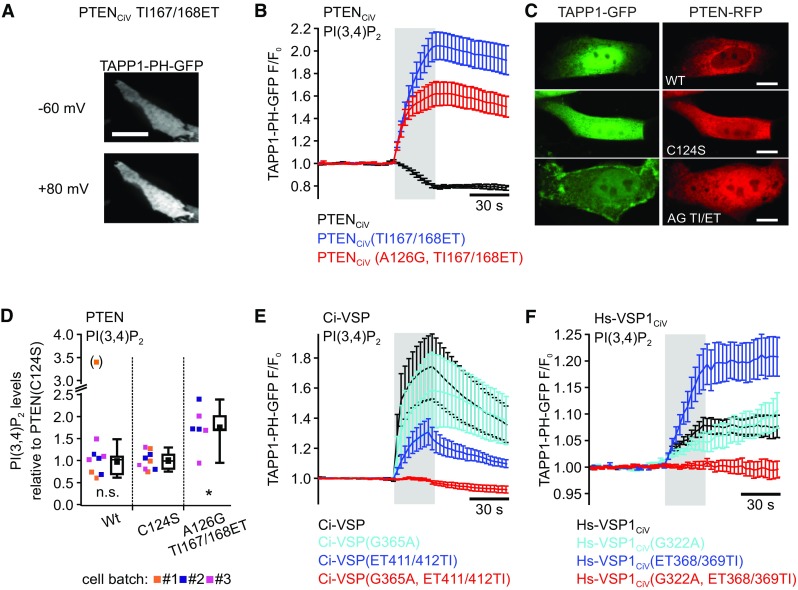
PI(3,4)P_2_ production through native PTEN, PTEN_CiV_ and VSP mutants demonstrates PI(3,4,5)P_3_-D5-phosphatase activity. **a** Representative TIRF image of a CHO cell co-expressing mRFP-PTEN_CiV_(TI167/168ET) and TAPP1-PH-GFP before (top) and during (bottom) depolarization of the cell membrane through whole-cell patch clamp (scale bar represents 10 μm). **b**, **e**, **f** Summarized TIRF signals in response to step depolarization from − 60 to + 80 mV (gray shading) of cells co-expressing the PI(3,4)P_2_ sensor TAPP1-PH-GFP with **b** wild type mRFP-PTEN_CiV_ (black trace, *n* = 6), mRFP-PTEN_CiV_(TI167/168ET) (blue, *n* = 7) or mRFP-PTEN_CiV_(A126G, TI167/168ET) (red, *n* = 8), **e** wild type mRFP-Ci-VSP (black, *n* = 7), mRFP-Ci-VSP(G365A) (cyan, *n* = 7), mRFP-Ci-VSP(ET411/412TI) (blue, *n* = 8), or mRFP-Ci-VSP(G365A, ET411/412TI) (red, *n* = 10), and **f** wild type mRFP-Hs-VSP1_CiV_ (black, *n* = 6), mRFP-Hs-VSP1_CiV_(G322A) (cyan, *n* = 6), mRFP-Hs-VSP1_CiV_(ET368/369TI) (blue, *n* = 7), or mRFP-Ci-VSP(G322A, ET368/369TI) (red, *n* = 7). **c** The panel shows representative confocal images of MDCK cells co-expressing TAPP1-PH-GFP together with wild type PTEN (top), PTEN(C124S) (middle), and PTEN(A126G, TI167/168ET) (bottom) (PTEN constructs were N-terminally tagged with RFP, all scale bars represent 10 µm; see Supplementary Fig. 2 for all analyzed cells). **d** PI(3,4)P_2_-levels were quantified by ELISA in independent triplets (three independent batches of PC-3 cells) expressing RFP-tagged soluble PTEN(wild type), PTEN(C124S), or PTEN(A126G, TI167/168ET). Note that for batch#1/wild type one outlier value was excluded from statistical analysis (marked with parentheses). PI(3,4)P_2_ concentrations obtained per batch and PTEN construct were normalized to the arithmetic mean calculated for the respective C124S-triplet. Individual (normalized) experiments are displayed together with summarized data (in boxplot representation: black-filled squares denote arithmetic means over all normalized values for each experimental condition). Statistical analysis was performed over all normalized values by two-sample *t* tests in comparison to PTEN(C124S): *n.s.* no statistical difference (PTEN(Wt)); *P ≤ 0.05 (PTEN(A126G. TI167/168ET)). Number of independent experiments: *n* = 3 for wild type and C124S, and *n* = 2 for the PTEN triple mutant)

For analysis of site-specific activity of the PTEN_CiV_ and VSPs mutants, we used TAPP1-PH-GFP to monitor PI(3,4)P_2_ levels at the cell membrane during voltage-dependent activation of the phosphatases (Fig. 3).

Upon activation of wild type PTEN_CiV_ by membrane potential depolarization, the fluorescence signal of TAPP1-PH-GFP measured with TIRF microscopy decreased due to dissociation of the sensor from the cell membrane (Fig. 3b). These results showed PI(3,4)P_2_ depletion through the chimeric enzyme, in line with previous studies on native PTEN [1–4] and PTEN_CiV_ [37].

In contrast to the wild type, in cells co-expressing PTEN_CiV_ carrying the three mutations A126G in the P loop and TI167/168ET in the TI loop depolarization of the membrane potential led to an increase in TAPP1-PH-GFP fluorescence at the membrane (Fig. 3b). This observation reflected PI(3,4)P_2_ production upon voltage-dependent activation of the enzyme. Taking into account that this triple mutant depleted PI(3,4,5)P_3_ (Fig. 2a), these findings characterized PTEN_CiV_(A126G, TI167/168ET) as a D5 phosphatase of PI(3,4,5)P_3_.

We next analyzed whether the triple mutation also changed the substrate specificity of soluble (native) PTEN not coupled to a voltage-sensor domain. To this end, we co-expressed PTEN (wild type and mutant) together with TAPP1-PH-GFP in MDCK cells and analyzed membrane association of the PI(3,4)P_2_ reporter with confocal microscopy (Fig. 3c; Fig. S2). TAPP1-PH-GFP was localized in the cytoplasm without any evident membrane association in all cells co-expressing wild type PTEN (42 cells analyzed in total; Fig. 3c and Fig. S2A). For catalytically inactive PTEN(C124S), membrane association of the PI(3,4)P_2_ reporter was observable in only 1 cell (36 cells analyzed; Fig. 3c and Fig. S2B). Markedly, we found clear membrane association of TAPP1-PH-GFP in approximately 37% of all cells co-expressing PTEN(A126G, TI167/168ET) (Fig. S2C). These findings demonstrated that expression of the PTEN triple mutant caused a pronounced increase in PI(3,4)P_2_ levels in the membrane of MDCK cells. Indeed, as quantified with PI(3,4)P_2_-mass ELISA (c.f. ref. [35]), we observed a significant increase in PI(3,4)P_2_ levels in PC-3 cells expressing PTEN(A126G, TI167/168ET) in comparison to cells expressing PTEN wild type or the catalytically dead mutant C124S (*P* ≤ 0.05; Fig. 3d). Thus, based on these results obtained with two independent cellular assays, we conclude that, just as for PTEN_CiV_, the three mutations in P and TI loop also conferred D5 site activity toward PI(3,4,5)P_3_ to native PTEN.

As such, D5 site-specific activity has also been described recently for the P loop mutant A126G in soluble PTEN and chimeric PTEN_CiV_ [35], a single amino acid exchange in the active site apparently was sufficient to confer D5 activity to PTEN. We thus decided to further analyze the significance of the TI/ET mutation. Remarkably, the TI167/168ET mutations alone also induced D5 site activity toward PI(3,4,5)P_3_ in PTEN_CiV_, as evident by an increase of TAPP1-PH-GFP fluorescence at the membrane, representing PI(3,4)P_2_ production during voltage-dependent activation (Fig. 3a, b). Thus, the A126G mutation in the P loop and the TI/ET exchange in the TI loop independently conferred “VSP-like” D5 site activity toward PI(3,4,5)P_3_ to the otherwise D3 site-specific phosphatase PTEN_CiV_.

Given the high degree of sequence similarity between VSPs and PTEN (Fig. S1), we next studied the effect of PTEN-like mutations in the P or the TI/gating loop of Ci-VSP and Hs-VSP1_CiV_. For both, the G/A exchange or the ET/TI mutations, TAPP1-PH-GFP translocated to the membrane during depolarization, similarly to translocation during activation of wild type VSPs (Fig. 3e, f). Since this fluorescence dynamics demonstrated production of PI(3,4)P_2_, we conclude that the pre-dominant D5 site activity of Ci-VSP and Hs-VSP1_CiV_ toward PI(3,4,5)P_3_ was not altered by introduction of these mutations individually. Of note—apart from the ET368/369TI mutations in Hs-VSP1_CiV_—the mutations in the P or TI/gating loop reduced phosphatase activity of Ci-VSP and Hs-VSP1_CiV_, as mirrored in the less pronounced membrane translocation of the PI(3,4)P_2_ probe TAPP1-PH-GFP during depolarization compared to the respective wild types enzymes (Fig. 3e, f).

When P and TI/gating loop mutations were both introduced into Ci-VSP and Hs-VSP1_CiV_, the fluorescence signal of TAPP1-PH-GFP remained constant upon activation of the enzymes (Fig. 3e, f) indicating no changes in the membrane PI(3,4)P_2_ concentration. In line with the absent activity of the triple mutant of Ci-VSP toward PI(3,4,5)P_3_ (c.f. Fig. 2b), these results also indicated lack of phosphatase activity of the VSP mutants carrying both, the P mutation and the TI/gating loop exchange.

In summary, individual and combined P and TI/gating loop mutations conferred VSP-like D5 site activity toward PI(3,4,5)P_3_ to PTEN_CiV_. In contrast, reciprocal mutations reduced or even abolished phosphatase activity of native VSPs.

### PTEN_CiV_(A126G, TI167/168ET) fully reproduces substrate specificity and site-specific activity of native VSPs

**Fig. 4:**
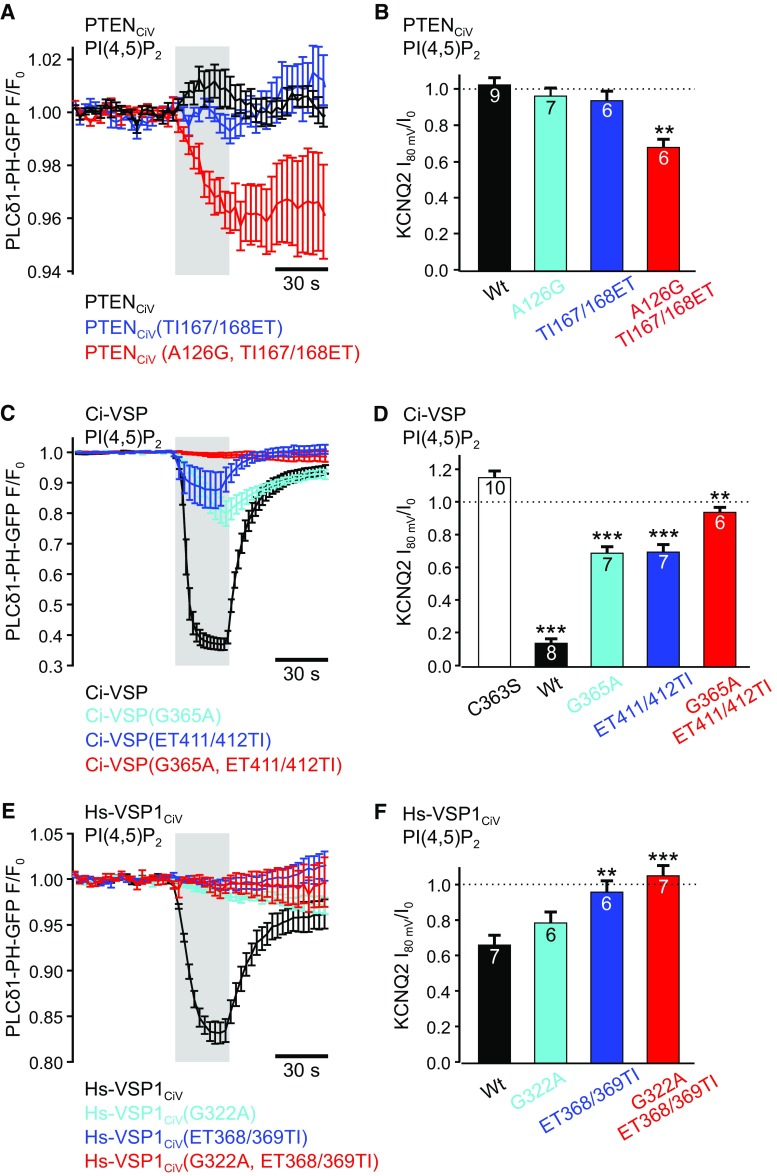
Depletion of PI(4,5)P_2_ by mutants of PTEN_CiV_ and VSPs demonstrates PI(4,5)P_2_-phosphatase activity. Summarized TIRF signals in response to step depolarization from − 60 to + 80 mV (gray shading) of cells co-expressing the PI(4,5)P_2_ sensor PLCδ_1_-PH-GFP and the indicated mRFP-tagged mutants of **a** PTEN_CiV_, **c** Ci-VSP, or **e** Hs-VSP1_CiV_. Summarized KCNQ2 currents in response to voltage-dependent activation of the indicated mutants of **b** PTEN_CiV_, **d** Ci-VSP, or **f** Hs-VSP1_CiV_. Holding potential was − 60 mV and phosphatases were activated at + 80 mV for 30 s. KCNQ2 currents were recorded at 0 mV. See Fig. S3 for voltage protocol and representative current recordings. **b** **P ≤ 0.01 indicates significant difference to PTEN_CiV_ wt. **d** **P ≤ 0.01 and ***P ≤ 0.001 indicate significant difference to catalytically dead mutant Ci-VSP(C363S). **f** **P ≤ 0.01 and ***P ≤ 0.001 indicate significant difference to Hs-VSP1_CiV_ wt. **a** Wild type mRFP-PTEN_CiV_ (black trace, *n* = 5), mRFP-PTEN_CiV_(TI167/168ET) (blue, *n* = 7) or mRFP-PTEN_CiV_(A126G, TI167/168ET) (red, *n* = 8), **c** wild type mRFP-Ci-VSP (black, *n* = 9), mRFP-Ci-VSP(G365A) (cyan, *n* = 5), mRFP-Ci-VSP(ET411/412TI) (blue, *n* = 11), or mRFP-Ci-VSP(G365A, ET411/412TI) (red, *n* = 8), and **e** wild type mRFP-Hs-VSP1_CiV_ (black, *n* = 6), mRFP-Hs-VSP1_CiV_(G322A) (cyan, *n* = 8), mRFP- Hs-VSP1_CiV_(ET368/369TI) (blue, *n* = 7), or mRFP-Ci-VSP(G322A, ET368/369TI) (red, *n* = 5)

Using PI(4,5)P_2_-specific PLCδ_1_-PH-GFP domain as biosensor, we then analyzed whether PTEN_CiV_ and VSP mutants exhibited D3 site activity toward PI(3,4,5)P_3_ or D5 site activity toward PI(4,5)P_2_. To this end, the TIRF-based assay using the fluorescently labeled PI(4,5)P_2_ binding domain was complemented with whole-cell patch clamp recordings of currents through KCNQ2 channels as an alternative PI(4,5)P_2_ sensor [48]. Control experiments with Ci-VSP showed robust inhibition of KCNQ2 currents (Fig. 4d, and Fig. S3A), demonstrating PI(4,5)P_2_ breakdown upon activation of Ci-VSP and, thus, applicability of our approach [49].

In cells co-expressing PTEN_CiV_, depolarization led to a slight increase in PLCδ_1_-PH-GFP fluorescence in the TIRF recordings (Fig. 4a), indicating PI(4,5)P_2_ production at the membrane. In combination with PTEN_CiV_-dependent PI(3,4,5)P_3_ depletion (Fig. 2a), these findings demonstrated that PTEN_CiV_ dephosphorylated PI(3,4,5)P_3_ at the D3 position.

In contrast, membrane binding of PLCδ_1_-PH-GFP was not affected by activation of PTEN_CiV_ carrying the TI loop mutations TI167/168ET indicating lack of phosphatase activity toward PI(4,5)P_2_ (Fig. 4a). In support of these findings, KCNQ2-mediated currents were also not reduced by activation of PTEN_CiV_(TI167/168ET) (Fig. 4b, and Fig. S3B). Since activation of this mutant caused accumulation of PI(3,4)P_2_ at the membrane (c.f. Fig. 3b) without altering PI(4,5)P_2_ concentrations (Fig. 4a, b), these findings demonstrated D5 site activity toward PI(3,4,5)P_3_, but not toward PI(4,5)P_2_ for PTEN_CiV_(TI167/168ET).

Similarly, we showed recently that the P loop mutation A126G changed the D3 site activity of PTEN_CiV_ to D5 site activity solely toward PI(3,4,5)P_3_ [35]. Here, we confirmed lack of PI(4,5)P_2_ activity of this mutant with electrophysiological recordings, as PTEN_CiV_(A126G) failed to inhibit KCNQ2 currents (Fig. 4b, and Fig. S3B). Thus, we conclude that PI(4,5)P_2_ did not serve as substrate of PTEN_CiV_(A126G).

Taken together, P and TI loop mutations independently conferred D5 site activity toward PI(3,4,5)P_3_, but not toward PI(4,5)P_2_ to PTEN_CiV_. Thus, these PTEN_CiV_ mutations did not fully recapitulate the phenotype of native VSPs.

When the mutations were introduced simultaneously into PTEN_CiV_ (A126G and TI167/168ET into P and TI loop, respectively), voltage-dependent stimulation caused a decrease in PLCδ_1_-PH-GFP fluorescence at the membrane (Fig. 4a) and substantially inhibited KCNQ2 currents (Fig. 4b and Fig. S3B). Both observations demonstrated PI(4,5)P_2_ depletion by PTEN_CiV_(A126G, TI167/168ET). Thus, these three amino acid exchanges in the active site as well as the TI loop, converted otherwise D3 specific PTEN_CiV_ into a D5 phosphatase that completely reproduced the pre-dominant substrate specificity of native VSPs.

### Mutations in P and gating loop reduce activity of native VSPs

In line with the pre-dominant D5 site phosphatase activity of native Ci-VSP and Hs-VSP1_CiV_ [23–28, 30], depolarization of the membrane potential decreased PLCδ_1_-PH-GFP membrane binding (Fig. 4c, e) and reduced KNCQ2 currents, when the wild type enzymes were co-expressed (Fig. 4d, f). When the G/A mutation was introduced into the P loop of these VSPs (G365A for Ci-VSP and G322A for Hs-VSP1_CiV_, Fig. 1b), no qualitative change in their PI(4,5)P_2_ D5 phosphatase activity was observed in the TIRF assay with PLCδ_1_-PH-GFP (Fig. 4c, e) or in the patch clamp recordings using KCNQ2 currents as PI(4,5)P_2_ sensors (Fig. 4d, f; Fig. S3A, C). Quantitatively, decrease in PLCδ1-PH-GFP fluorescence was reduced and inhibition of KCNQ2 currents was attenuated for Ci-VSP(G365A) in comparison to the wild type enzyme (Fig. 4c, d). Upon activation of Hs-VSP1_CiV_(G322A), only minor changes in PLCδ1-PH-GFP fluorescence were detected (Fig. 4e). However, the mutant still depleted PI(4,5)P_2_ as demonstrated by inhibition of KCNQ2-mediated currents (Fig. 4f), although channel inhibition was reduced compared to wild type Hs-VSP1_CiV_.

For the gating loop mutations ET411/412TI in Ci-VSP, membrane dissociation of PLCδ_1_-PH-GFP and inhibition of KCNQ2 currents were still observable (Fig. 4c, d). These results demonstrated PI(4,5)P_2_ consumption by this mutant, although phosphatase activity was reduced compared to the wild type enzyme. In contrast, we did not detect changes in PI(4,5)P_2_ levels upon activation of Hs-VSP1_CiV_(ET368/369TI) (Fig. 4e, f and Fig. S3C).

As for the gating loop mutant, for the triple Hs-VSP1_CiV_(G322A, ET368/369TI) mutant, voltage-dependent reduction in PI(4,5)P_2_ concentration was not detectable (Fig. 4e, f). In contrast, Ci-VSP(G365A, ET411/412TI) exhibited residual activity toward PI(4,5)P_2_ as seen in minute translocation of PLCδ_1_-PH-GFP (Fig. 4c) and in a slight, albeit significant inhibition of KCNQ2 currents (Fig. 4d, Fig. S3A).

Taken together, insertion of PTEN-like mutations into the P and gating loop of native VSPs reduces or even abolished PI(4,5)P_2_ phosphatase activity of Ci-VSP and Hs-VSP1_CiV_.

## Discussion

Despite high similarity in sequence and structure, PTEN and VSPs differ in their reaction specificity. The reason for this discrepancy is not known. In this study, we shed light on this question by systematically exchanging amino acids in the active site motif (P loop) and the TI or “gating loop” that both form parts of the substrate binding pocket. Our results (summarized in Table 1) provide insights into the structural origin of the substrate specificity of PTEN: (i) The exchange of the TI loop signature motif with the ET pair of VSPs changed phosphatase activity of PTEN_CiV_ from D3- to a VSP-like D5 site specificity toward PI(3,4,5)P_3_. (ii) In PTEN_CiV_, combining TI/ET loop mutations with the alanine-to-glycine exchange in the P loop, removing major sequence differences to native VSPs, induced D5 site activity toward both, PI(3,4,5)P_3_ and PI(4,5)P_2_. Thus, the PTEN_CiV_ triple mutant fully reproduced the phosphatase phenotype of Ci-VSP. Importantly, our results unequivocally demonstrated that the same amino acid residues also determine substrate specificity of native (soluble) PTEN. (iii) In contrast, reciprocal mutations in Ci-VSP and Hs-VSP1_CiV_ did not alter substrate specificity, but reduced or even abolished their phosphatase activity.

**Table 1:** Summary of observed enzymatic activities of PTEN_CiV_ and VSPs mutants

Substrate	PI(3,4,5)P_3_		PI(3,4)P_2_	PI(4,5)P_2_
Position	D3	D5	D3	D5
PTEN_CiV_	+	−	+	−
PTEN_CiV_(A126G)	−	+	−	−
PTEN_CiV_(TI167/168ET)	−	+	−	−
PTEN_CiV_(A126G,TI167/168ET)	−	+	−	+
VSP	−	+	−	+
VSP(G/A)	−	+	−	+
VSP(ET/TI)	−	+	−	+
VSP(G/A, ET/TI)	−	−	−	−

‘+’ indicates activity, ‘−’ indicates lack thereof. Since the activity of Hs-VSP1_CiV_ is qualitatively identical to that of Ci-VSP, both VSPs have been summarized. ‘G/A’ refers to ‘G365A’ for Ci-VSP and ‘G322A’ for Hs-VSP1_CiV_, ‘ET/TI’ to ‘ET411/412TI’ for Ci-VSP and ‘ET368/369TI’ for Hs-VSP1. Information on PTEN_CiV_(A126G) is taken from Costa et al. [35]

### PTEN_CiV_ is an established tool to study substrate specificity of native PTEN

Recently, we engineered chimeric voltage-sensitive PTEN_CiV_ by fusing the voltage sensor of Ci-VSP to the catalytically active domain of PTEN (originally named “CiVSPTEN16”) [37]. We could demonstrate that this chimeric enzyme is rapidly switched on by depolarizing the membrane potential precisely reproducing the well-known D3 site phosphatase activity of native PTEN [35, 37, 42]. Furthermore, we here directly show that mutations in gating and TI loops change substrate specificity of native PTEN and PTEN_CiV_ precisely the same way. Thus, PTEN_CiV_ is a suitable tool to study catalytic mechanisms of the tumor suppressor PTEN. Importantly, substrate specificity of the enzyme can easily be monitored in living cells with the combination of TIRF microscopy and patch clamp technique that is well-established for studies of VSPs [23, 25, 37]. This approach entails two remarkable advantages: (i) Direct and rapid control of phosphatase activity by membrane voltages enables the exact differentiation of cellular responses before, during and after the catalytic reaction. Consequently, changes in concentrations of discrete PI species at the plasma membrane can be determined with high spatiotemporal precision to characterize substrate specificity of the phosphatase. (ii) Chimeric enzymes allow for characterization of phosphatase activity in native lipid environments. Artificial membrane systems or vesicles, which are normally used to study phosphatase activity of PTEN and its homologs in vitro, hardly mimic the complex lipid composition of cellular membranes. In particular, it is well-established that with phosphatidylserine and PI(4,5)P_2_, two lipids crucially regulate phosphatase activity of PTEN, either by mediating membrane binding of the enzyme or by allosterically activating the catalytic reaction [3, 50–55]. Thus, results obtained in vitro and in cellular assays frequently diverge. As a case in point, Hs-VSP1 was originally described as PI phosphatase with PTEN-like D3 site substrate specificity in vitro [21]. Taking into account that Hs-VSP1 shares higher sequence homology in P and gating loop to D5 site phosphatases (such as Ci-VSP) than to PTEN (Fig. 1b and Fig. S1), the claim of D3 site specificity was somewhat surprising. Finally, studies on Hs-VSP1 utilizing the Hs-VSP1_CiV_ chimera in living cells that allow for measurements under more physiological conditions demonstrated its actual VSP-like D5 site activity [23].

Therefore, we conclude that artificial membrane systems are well suitable to detect phosphatase activity of PTEN and its homologs in general, but cellular membranes are better suited to study the substrate specificity of these phosphatases in their native lipid environment.

### Determinants of substrate specificity of PTEN

In PTEN and VSPs, at least three loops (P, TI/gating and WPD loop) form the substrate binding pocket (Fig. 1c). With less than 1 Å deviation in the Cα trace of the protein backbone, the P loop shows only minor structural differences among the phosphatases [27, 34] and differs only in one alanine (PTEN) to glycine (VSPs) exchange (Fig. 1b). The A126G exchange shifts substrate specificity of PTEN_CiV_ from pure D3 to D5 site activity toward PI(3,4,5)P_3_ and abolishes activity toward PI(3,4)P_2_ [35]. Here, we confirmed that PTEN_CiV_(A126G) is not active toward PI(4,5)P_2_ using KCNQ2 channels as read-out. These findings indicate that the alanine in the active site motif might be crucial for establishing D3 site activity in PTEN, but however additional structural properties of the substrate binding pocket may be required to establish full D5 phosphatase activity of native VSPs in PTEN.

Indeed, the canonic TI pair has already been suggested to play a role in substrate binding of PTEN. Lee et al. considered T167 as important for positioning a PI(3,4,5)P_3_ molecule into the active site, since they observed close vicinity between this residue and the D4/D5 sites of Ins(1,3,4,5)P_4_ in an X-ray-based structural model [32]. Hypothetically, these interactions could stabilize the binding between the D3 site of PI(3,4,5)P_3_ and the catalytic residue C124. Thus, mutating T167 might alter PI(3,4,5)P_3_ binding in such a way that the D5 site is no longer stabilized by the TI pair, so that it can bind to the catalytic center. Furthermore, homologous positions of the TI pair in other PTPs, including VSPs, were shown to interfere with catalysis [31]. Mutating TI167/168 into their VSP counterpart ET readily converted PTEN_CiV_ into a PI(3,4,5)P_3_-D5 site-specific phosphatase with no activity toward PI(3,4)P_2_ and PI(4,5)P_2_, which reproduces the phenotype of PTEN_CiV_(A126G). Thus, the P and TI loop mutations independently determine site specificity of PTEN_CiV_ toward PI(3,4,5)P_3_, but did not allow for VSP-like PI(4,5)P_2_-D5 site activity. Only the combination of P and TI loop mutations produced the full phenotype of VSPs enabling dephosphorylation activity toward the D5 site of both substrates, PI(3,4,5)P_3_ and PI(4,5)P_2_. Noteworthy, the same combination of mutations also changed the substrate specificity of native (soluble) PTEN demonstrating that the same residues determine substrate specificity of the tumor suppressor in living cells. Based on our results, we conclude that A126 and TI167/168 are determinants for the substrate specificity in PTEN, whereby simultaneous mutations on both sites additively affect enzymatic activity.

### The role of the P loop glycine for the phosphatase activity of VSPs

Sequence comparison reveals that all known VSPs possess a glycine in their HCX_5_R motif, whereas PTEN shows an alanine at this position (Fig. 1b). Thus, it was tempting to speculate that the glycine determines VSP-like D5 site activity toward PI(3,4,5)P_3_ and PI(4,5)P_2_ [20, 23–28, 30], whereas the alanine is crucial for D3 site activity. Indeed, findings of Iwasaki et al. supported this hypothesis [24], since they observed pronounced activity of Ci-VSP(G365A) toward PI(3,4)P_2_ in in vitro assays using isolated catalytic domains.

Upon activation of Ci-VSP(G365A) and Hs-VSP1_CiV_(G322A), we detected a reliable increase in TAPP1-PH-GFP binding to the membrane indicating pre-dominant D5 site activity toward PI(3,4,5)P_3_ and comparably low activity toward PI(3,4)P_2_. These findings are in line with previous studies by Kurokawa et al. [26] and Liu et al. [27], who independently characterized Ci-VSP(G365A) with fluorescent PIP probes in *Xenopu*s *laevis* oocytes. Thus, in vitro results most probably do not represent substrate specificity of VSPs in living cells.

Additionally, Iwasaki et al. concluded absence of PI(4,5)P_2_ activity for Ci-VSP(G365A) based on experiments performed on *Xenopus laevis* oocytes with two PI(4,5)P_2_ reporter systems, GIRK2 (Kir3.2) channels and the GFP-tagged PLCδ_1_-PH domain [24]. In contrast, we here demonstrate reduced, but not substantially altered activity of Ci-VSP(G365A) against PI(4,5)P_2_. A straightforward explanation for the different results is that the residual activity of this Ci-VSP mutant was too minute to be detectable with the methods employed by Iwasaki et al.: (i) Whereas Iwasaki and colleagues monitored membrane association of PLCδ_1_-PH-GFP with confocal microscopy, we used TIRF imaging that is apparently more sensitive for the detection of small changes in membrane-bound fluorescence. (ii) Iwasaki et al. used a holding potential of 0 mV to activate Ci-VSP, whereas we applied + 80 mV resulting in stronger activation of Ci-VSP. Indeed, we showed earlier [25] that activation of Ci-VSP at 0 mV caused markedly less decrease in membrane binding of the PLCδ_1_-PH probe than activation at + 80 mV (approximately 10 vs. 80% of maximal effect). (iii) Given their low PI(4,5)P_2_ affinity, KCNQ2 (K_v_7.2) channels are highly sensitive to PI(4,5)P_2_ depletion [56]. Thus, KCNQ2 channels most probably constitute more sensitive PI(4,5)P_2_ sensors than GIRK2 channels utilized by Iwasaki et al.. This sensitivity is reflected in the work by Rjasanow et al. [49] showing that KCNQ2 currents were reduced by about 50% when Ci-VSP was activated at a holding potential of − 20 mV compared to members of the GIRK channel family that needed + 10 mV (Kir1.1) to + 30 mV (Kir2.1) for comparative inhibition. Thus, GIRK2 channels might be too insensitive to detect the minute PI(4,5)P_2_ depletion that is caused by Ci-VSP(G365A) at 0 mV.

In summary, we conclude that the glycine-to-alanine exchange in the P loop does not convert VSPs into D3 site specific phosphatases, but attenuates their pre-dominant D5 site activity. It can be speculated that the bulkier side chain of alanine reduces the size of the substrate binding pocket in the VSP(G/A) mutants and, therefore, hampers PI substrate docking into the active site.

### The role of the ET pair for determining substrate specificity of VSPs

Given significant variations in sequence and length between PTEN and VSPs (Fig. S1), the TI/gating loop might determine substrate specificity of the phosphatases. Crystallographic data of the cytosolic fragment of Ci-VSP [27, 34], and molecular dynamics simulations of the full-length Ci-VSP protein with its voltage sensor embedded in a lipid bilayer [36] suggested that this gating loop may adopt different conformations, with the glutamate in the ET pair changing its position to open or close the active site [27]. However, results obtained with Ci-VSP(E411) mutants are inconsistent. Using isolated cytosolic fragments in a malachite green-based phosphatase assay in vitro, Matsuda et al. observed no significant differences in phosphatase activity and substrate specificity between wild type Ci-VSP and E411 mutants, carrying either hydrophobic (E411A) or polar mutations (E411Q and E411T) [34]. In contrast, Liu et al. detected an increase in D5 site activity of Ci-VSP(E411T) toward PI(3,4,5)P_3_ using confocal microscopy on *Xenopus* laevis oocytes which co-expressed full-length Ci-VSP together with the PI(3,4)P_2_ sensor TAPP1-PH-GFP [27].

**Fig. 5:**
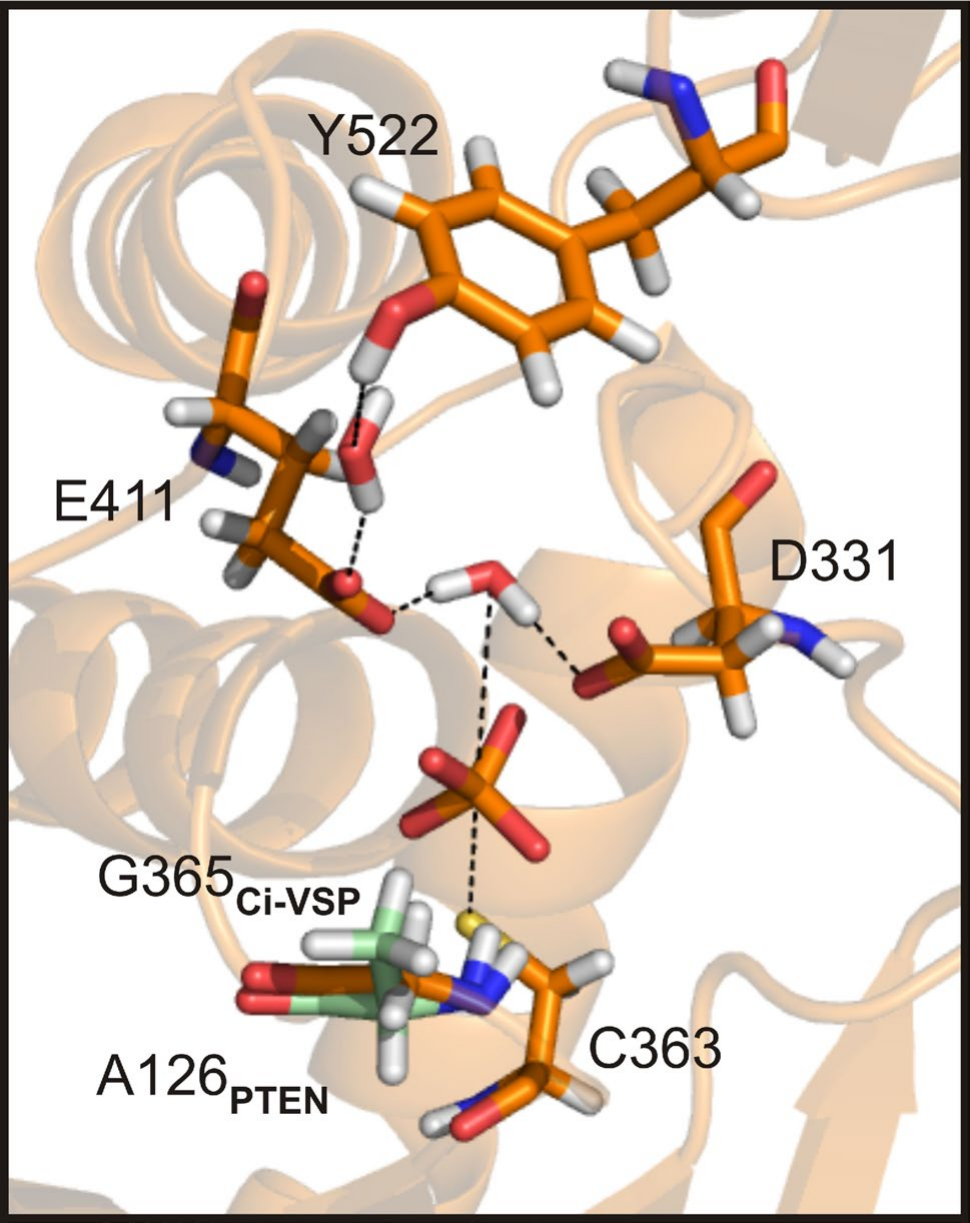
Hydrogen bonds in the substrate binding pocket of Ci-VSP. View into the substrate binding pocket of Ci-VSP. Residues are depicted as sticks: C363 and G365 in the active site motif/P loop, D331 in the WPD loop, E411 in the gating loop, and Y522 in the CBR3-loop of the C2-domain. The structure of Ci-VSP was obtained by MD simulation as described before [31, 36]. Structural alignment of the Ci-VSP-model with the 3D-structure of PTEN (PDB 1d5r, [32]) revealed the position of A126 (in green) being homologous to G365 in Ci-VSP. Hydrogen bonds to water molecules and a phosphate ion in the active site are indicated as black dotted lines

Interestingly, Liu et al. described that insertion of hydrophobic residues at position E411 (Ile, Leu, or Phe) reduced phosphatase activity of Ci-VSP toward PI(3,4,5)P_3_ and PI(4,5)P_2_, whereas hydrophilic side-chains (Asn, Gln, or Thr) were more tolerable for phosphatase activity [27]. Fully in line with our results on ET/TI mutants, Liu et al. suggested that the hydrophilic character of the E411 side chain was important to maintain enzymatic activity of VSPs. As proposed earlier [31], the glutamate at position E411 in the ET pair in the gating loop might directly participate in the catalytic reaction of Ci-VSP by forming hydrogen bonds to the water molecule required for substrate hydrolysis (Fig. 5). Such a network of hydrogen bonds was already described for several members of the PTP superfamily (e.g., PTP1B [57, 58] and YopH [59, 60]). Mutating the ET pair of VSPs to the less charged TI motif putatively destabilizes these hydrogen bonds in the substrate binding pocket and, therefore, causes the reduction in phosphatase activity. Even more, in the VSP triple-mutants, the impaired electrostatic interactions of the ET/TI pair could be further accompanied by the spatial constraints introduced with the G/A mutation in the P loop (Fig. 5), leading to the dramatic reduction in phosphatase activity.

### Different results of PTEN_CiV_ and VSPs provide insight into structures crucial for phosphatase activity in VSPs

Why is PTEN’s substrate specificity readily changed by three simple point mutations, whereas Ci-VSP and Hs-VSP1_CiV_ were immune to the reciprocal mutations? A straightforward answer to this question is that certain intramolecular structures may contribute to the substrate binding pocket in native VSPs that are absent or different in PTEN. But what additional structural features could determine the substrate specificity in VSPs?

Taking 3D structures of PTEN [32, 33] and Ci-VSP [27, 34] into account, the major structural difference between these phosphatases is the orientation of the CBR3 loop in the C2 domain (Fig. 1c), with a tyrosine at position 522 (Y522) pointing into the substrate binding pocket in Ci-VSP. This tyrosine is well-conserved among VSPs, but missing in PTEN (Fig. S1). Previous studies on Ci-VSP suggested a catalytic role for Y522 [27, 28], because mutating it into phenylalanine qualitatively preserved substrate specificity of Ci-VSP, but shifted the apparent voltage dependence of phosphatase activity to depolarized potentials. Because voltage-clamp fluorometry further showed a shift in voltage dependence of voltage sensor motion to higher voltages, Castle et al. suggested that the Y522F-mutation increases the energy barrier of the protein for entering the catalytic cycle [28]. Along these lines, it can be speculated that the mutations we introduced here in VSPs might interfere with steric constraints imposed by the tyrosine in the CBR3 loop and, therefore, prohibit proper substrate binding and hydrolysis. MD simulations on Ci-VSP [31, 36] indeed predict interactions between E411 and Y522 through sharing hydrogen bonds to the same water molecule (Fig. 5). Since these interactions might stabilize the position of E411 in the substrate binding pocket, the CBR3 loop could act as an additional structure that influences substrate specificity in VSPs. However, preliminary data demonstrated that activation of Ci-VSP(Y522A) also results in PI(3,4)P_2_ accumulation at the membrane, reproducing wild type activity (Fig. S4). Introducing this mutation in the inactive triple mutant of Ci-VSP neither rescued D5 site activity toward PI(3,4,5)P_3_ nor induced PTEN-like D3 site activity toward PI(3,4)P_2_ in Ci-VSP (Fig. S4). Therefore, distinct effects of TI/ET mutations on the phosphatase activity of PTEN and VSPs cannot be explained by the contribution of the CBR3 loop tyrosine to the substrate binding pocket in VSPs. Unfortunately, no structural data exist so far about PTEN or VSPs with a PI substrate molecule that is bound to the active site. Thus, further effort is needed to reveal the orientation of single residues in the active site that explain the differences in substrate specificity between PTEN and VSPs.

### Implications for the role of PTEN as tumor suppressor

PTEN is one of the most frequently disrupted tumor suppressors in cancer [6, 8, 9] highlighting that, in most cases, loss-of-function mutations of PTEN contribute to tumor genesis and development of cancer. Recently, we identified an alanine-to-glycine mutation in the active site of PTEN in a prostate tumor sample of a cancer patient [35]. Using a similar approach as presented here, we demonstrated that this mutation induced a gain-of-function of phosphatase activity, converting PTEN from a PI(3,4,5)P_3_ D3 phosphatase into a D5 phosphatase. This alteration of enzymatic specificity caused an oncogenic increase in PI(3,4)P_2_ levels thereby potentially affecting tumor migration and metastasis. In the present study, we provide additional evidence for conversion of PTEN´s site-specific activity through seemingly minor amino acid exchanges in the substrate binding pocket. Therefore, other PTEN mutations may also exhibit oncogenic potential through similar or distinct gain-of-function. Different functional outcomes in terms of catalytic activity may promote tumor genesis or progression through diverging molecular mechanisms, which should be considered in thinking toward prospective PTEN-targeted, patient-specific therapy. Along these lines, the characterization of substrate specificity of tumor-specific PTEN mutants in living cells seems prudent. To this end, employing engineered voltage-gated PTEN chimeras may provide a useful strategy. It should be mentioned here, that we have recently presented a technically simplified approach that is easily scalable and, therefore, may be suitable for testing mutant-specific pharmacological interventions [35, 37, 42]. In particular, the PTEN_CiV_-mutants A126G and TI167/168ET, we described here, might be helpful enzymatic machineries for studies on particular PI-associated pathways, since they allow for manipulation of PI(3,4,5)P_3_ while producing PI(3,4)P_2_ without disturbing membrane PI(4,5)P_2_ levels. Presumably, more advanced knowledge of the determinants of the substrate specificity might enable the design of voltage-sensitive phosphatases with desirable substrate specificities expanding their applicability for studies on PI signaling.

## Electronic supplementary material

Below is the link to the electronic supplementary material.

Supplementary material 1 (PDF 11552 kb)
