# Supplementary material for: A126 in the active site and TI167/168 in the TI loop are essential determinants of the substrate specificity of PTEN
Source: Cell Mol Life Sci. 2018 Jul 9;75(22):4235–50. doi: 10.1007/s00018-018-2867-z (PMC6182344; doi:10.1007/s00018-018-2867-z)
Supplement: Supplementary file 1 — Supplementary material 1 (PDF 11552 kb) [file 18_2018_2867_MOESM1_ESM.pdf]

```
Ci-VSP        MEGFDGSDFSPPADLVGVDGAVMRNVV-DVTINGD-VTAPPKAAPRKSESVKKVHWNDVDQGPSEKPETRQE   70
Hs-VSP1       ----------------------MNESPQTNEFKGTTEEAPAKESPHTSEFKGAALV-----SPISK------   39
Hs-VSP1civ    MEGFDGSDFSPPADLVGVDGAVMRNVV-DVTINGD-VTAPPKAAPRKSESVKKVHWNDVDQGPSEKPETRQE   70
PTENciv       MEGFDGSDFSPPADLVGVDGAVMRNVV-DVTINGD-VTAPPKAAPRKSESVKKVHWNDVDQGPSEKPETRQE   70
PTEN          ------------------------------------------------------------------------

Ci-VSP        ERIDIPEISGLWWGENEHGVDDGRMEIPTTGVGRVQFRVRAVIDHLGMRVFGVFLIFLDIILMIIDLSLPGK   142
Hs-VSP1       ---------SMLERLSKFEVEDA--ENVASYDSKIKKIVHSIVSSFAFGIFGVFLVLLDVTLLLADLIFTDS   100
Hs-VSP1civ    ERIDIPEISGLWWGENEHGVDDGRMEIPTTGVGRVQFRVRAVIDHLGMRVFGVFLIFLDIILMIINLSLPGK   142
PTENciv       ERIDIPEISGLWWGENEHGVDDGRMEIPTTGVGRVQFRVRAVIDHLGMRVFGVFLIFLDIILMIIDLSLPGK   142
PTEN          ------------------------------------------------------------------------

Ci-VSP        SESSQSFYDGMALALSCYFMLDLGLRIFAYGPKNFFTNPWEVADGLIIVVTFVVTIFYTVLDEYVQETGADG   214
Hs-VSP1       KLYIPLEYRSISLAIGLFFLMDVLLRVFVEGRQQYFSDLFNILDTAIIVIPLLVDVIYIFFDIKLLRN-IPR   171
Hs-VSP1civ    SESSQSFYDGMALALSCYFMLDLGLRIFAYGPKNFFTNPWEVADGLIIVVTFVVTIFYTVLDEYVQETGADG   214
PTENciv       SESSQSFYDGMALALSCYFMLDLGLRIFAYGPKNFFTNPWEVADGLIIVVTFVVTIFYTVLDEYVQETGADG   214
PTEN          ------------------------------------------------------------------------

Ci-VSP        LGRLVVLARLLRVVRLARIFYSHQQMKASSRRTISQNKRRYRKDGFDLDLTYVTDHVIAMSFPSSGRQSLFR   286
Hs-VSP1       WTHLVRLLRLIILIRIFHLLHQKRQLEKLMRRLVSENKRRYTRDGFDLDLTYVTERIIAMSFPSSGRQSFYR   243
Hs-VSP1civ    LGRLVVLARLLRVVRLARIFYSHQQLEKLMRRLVSENKRRYTRDGFDLDLTYVTERIIAMSFPSSGRQSFYR   286
PTENciv       LGRLVVLARLLRVVRLARIFYSHQQMKASSRRTISQNKRRYQEDGFDLDLTYIYPNIIAMGFPAERLEGVYR   286
PTEN          -------------------------MTAIIKEIVSRNKRRYQEDGFDLDLTYIYPNIIAMGFPAERLEGVYR   47

Ci-VSP        NPIGEVSRFFKTKHPDKFRIYNLCSERGYDETKFDNHVYRVMIDDHNVPTLVDLLKFIDDAKVWMTSDPDHV   358
Hs-VSP1       NPIEEVVRFLDKKHRNHYRVYNLCSERAYDPKHFHNRVSRIMIDDHNVPTLHEMVVFTKEVNEWMAQDLENI   315
Hs-VSP1civ    NPIEEVVRFLDKKHRNHYRVYNLCSERAYDPKHFHNRVSRIMIDDHNVPTLHEMVVFTKEVNEWMAQDLENI   358
PTENciv       NNIDDVVRFLDSKHKNHYKIYNLCAERHYDTAKFNCRVAQYPFEDHNPPQLELIKPFCEDLDQWLSEDDNHV   358
PTEN          NNIDDVVRFLDSKHKNHYKIYNLCAERHYDTAKFNCRVAQYPFEDHNPPQLELIKPFCEDLDQWLSEDDNHV   119
                                                      WPD loop

Ci-VSP        IAIHCKGGKGRTGTLVSSWLLEDGKFDTAKEALEYFGSRRTDFEVGDVFQGVETASQIRYVGYFEKIKKNYG   430
Hs-VSP1       VAIHCKGGKGRTGTMVCALLIASEIFLTAEESLYYFGERRTNKTHSNKFQGVETPSQNRYVGYFAQVKHLYN   387
Hs-VSP1civ    VAIHCKGGKGRTGTMVCALLIASEIFLTAEESLYYFGERRTNKTHSNKFQGVETPSQNRYVGYFAQVKHLYN   430
PTENciv       AAIHCKAGKGRTGVMICAYLLHRGKFLKAQEALDFYGEVRTRDK-----KGVTIPSQRRYVYYYSYLLKNHL   425
PTEN          AAIHCKAGKGRTGVMICAYLLHRGKFLKAQEALDFYGEVRTRDK-----KGVTIPSQRRYVYYYSYLLKNHL   186
                  P loop                                    TI loop

Ci-VSP        GQLPPMKKLKVTGVTITAIQGVGRGNGSDLSMQIVSERQEVLLCKFAEGYNCALQYDATDDCVTCEVKNCPV   502
Hs-VSP1       WNLPPRRILFIKRFIIYSIR----GDVCDLKVQVVMEKKVVFS--STSLGNCSILHDIETDKILINVYDGPP   453
Hs-VSP1civ    WNLPPRRILFIKRFIIYSIR----GDVCDLKVQVVMEKKVVFS--STSLGNCSILHDIETDKVLINVYDGPP   496
PTENciv       -DYRPVALLFHKMM-FETI-PMFSGGTCNPQFVVCQLKVKIYSSNSG-------PTRREDKFMYFEFPQPLP   487
PTEN          -DYRPVALLFHKMM-FETI-PMFSGGTCNPQFVVCQLKVKIYSSNSG-------PTRREDKFMYFEFPQPLP   248

Ci-VSP        LAGDIKVRFMSTSKSLPRGYDNCPFYFWFNTSLVEGDH---------------------------------V   541
Hs-VSP1       LYDDVKVQFFS--SNLPKYYDNCPFFFWFNTSFIQNNR---------------------------------L   490
Hs-VSP1civ    LYDDVKVQFFS--SNLPKYYDNCPFFFWFNTSFIQNNR---------------------------------L   533
PTENciv       VCGDIKVEFFHKQNKMLK--KDKMFHFWVNTFFIPGPEETSEKVENGSLCDQEIDSICSIERADNDKEYLVL   557
PTEN          VCGDIKVEFFHKQNKMLK--KDKMFHFWVNTFFIPGPEETSEKVENGSLCDQEIDSICSIERADNDKEYLVL   318
                             CBR3 loop

Ci-VSP        TLKREEIDNPHKKKTWKIYRDNFTVKLTFSDAEDI-------------------------------------   576
Hs-VSP1       CLPRNELDNPHKQKAWKIYPPEFAVEILFGEK----------------------------------------   522
Hs-VSP1civ    CLPRNELDNPHKQKAWKIYPPEFAVEILFGKK----------------------------------------   565
PTENciv       TLTKNDLDKANKDKANRYFSPNFKVKLYFTKTVEEPSNPEASSSTSVTPDVSDNEPDHYRYSDTTDSDPENE   629
PTEN          TLTKNDLDKANKDKANRYFSPNFKVKLYFTKTVEEPSNPEASSSTSVTPDVSDNEPDHYRYSDTTDSDPENE   390

Ci-VSP        -------------      576
Hs-VSP1       -------------      522
Hs-VSP1civ    -------------      565
PTENciv       PFDEDQHSQITKV      642
PTEN          PFDEDQHTQITKV      403
```

Supplemental Figure 1. Complete alignment of studied phosphatases and VSPs.

Supplemental Figure 1. Complete sequence alignment of studied phosphatases and VSPs.

Sequence alignment of Ci-VSP, Hs-VSP1, Hs-VSP1$_{CiV}$, PTEN$_{CiV}$ and PTEN as performed with ClustalX 2.1 [1] (UniProt accession numbers are: Q6XPS3, Hs-VSP1, also known as "TPTE2" or "TPIP"; P60484, human PTEN; Q4W8A1, Ci-VSP). N-terminus and voltage-sensor domain of Ci-VSP fused to the catalytic domain of Hs-VSP1 and PTEN are highlighted in *green* in the chimeras Hs-VSP1$_{CiV}$ and PTEN$_{CiV}$. Please note that Hs-VSP1$_{CiV}$(D136N) mutant (termed Hs-VSP1$_{CiV}$ or wild type) was used throughout this study (see also "Materials and Methods"). The D136N-mutation is highlighted in *magenta* in the alignment. Amino acids in loop regions forming substrate binding pocket (P, TI/ gating loop and WPD loop) are colored in *red*. Positions in P and TI/gating loop studied in this work are highlighted in *yellow*. Amino acids of CBR3-loop in the C2-domain are colored in *brown* with tyrosine (numbered Y522 in Ci-VSP) highlighted in *cyan*.

[1] Larkin MA, Blackshields G, Brown NP, et al (2007) Clustal W and Clustal X version 2.0. Bioinformatics 23:2947–2948 . doi: 10.1093/bioinformatics/btm404

Supplemental Figure 2A. MDCK cells co-expressing RFP-PTEN(WT) together with TAPP1-PH-GFP.

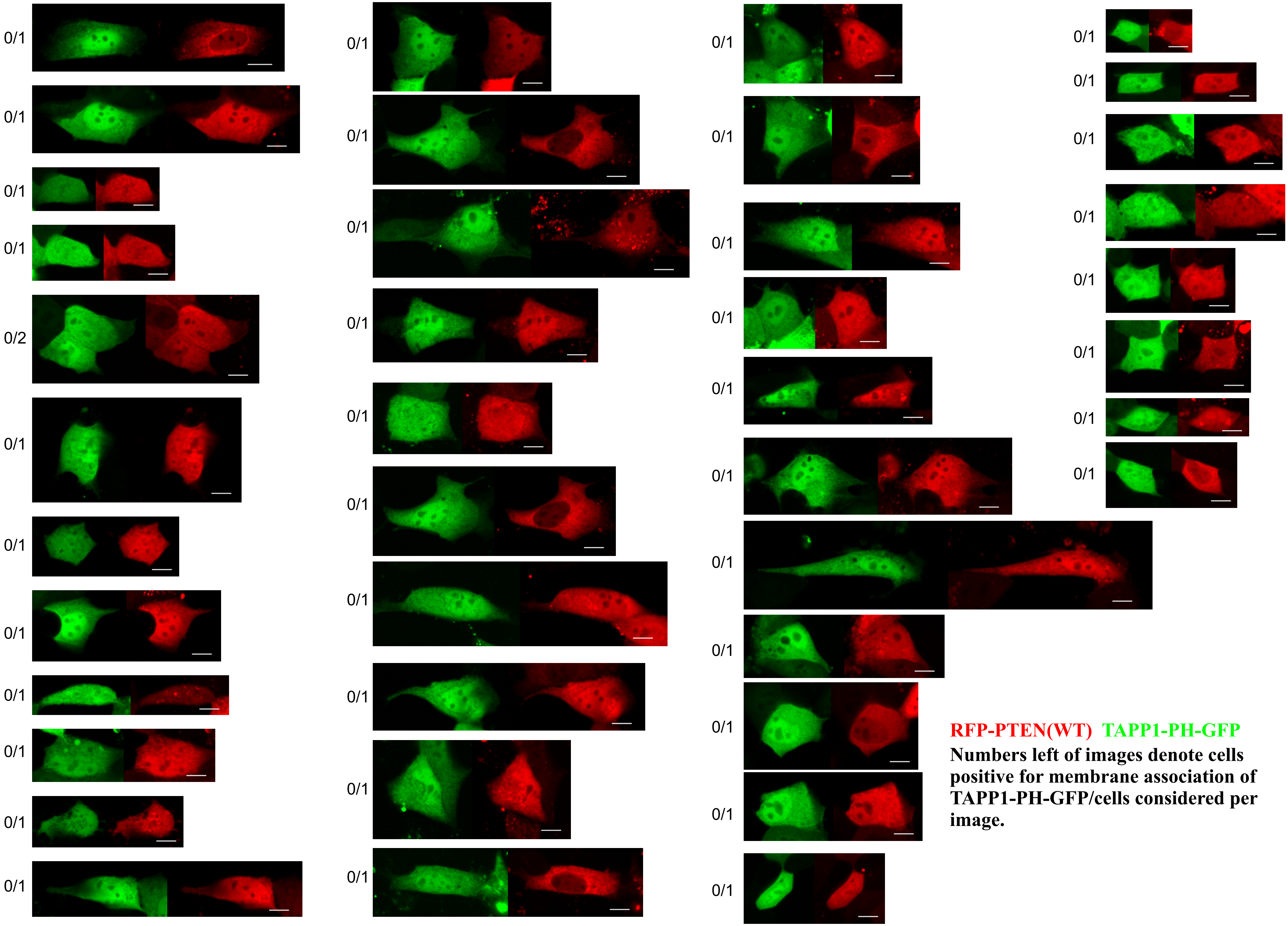

RFP-PTEN(WT) TAPP1-PH-GFP

Numbers left of images denote cells positive for membrane association of TAPP1-PH-GFP/cells considered per image.

Supplemental Figure 2B. MDCK cells co-expressing RFP-PTEN(C124S) together with TAPP1-PH-GFP.

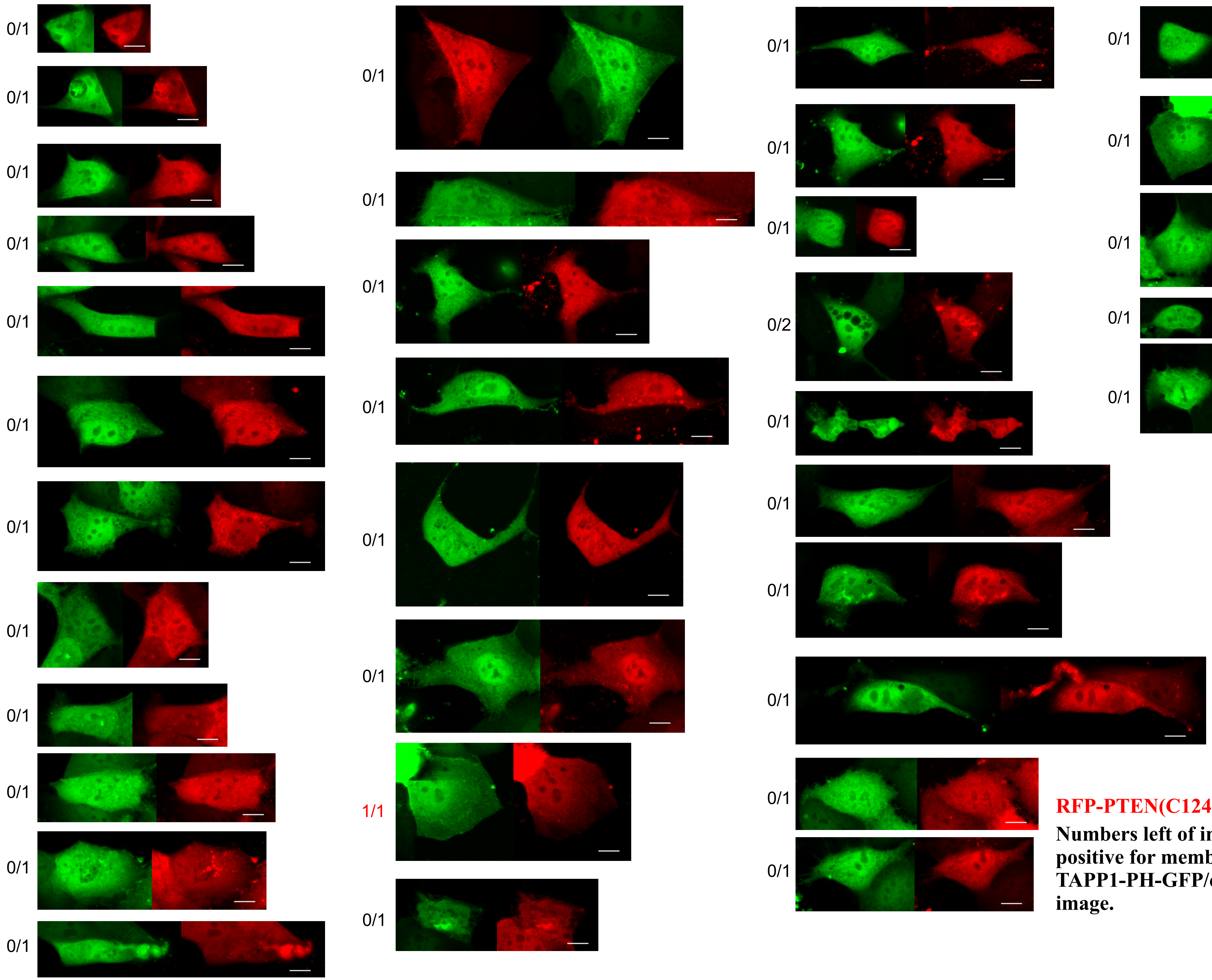

RFP-PTEN(C124S) **TAPP1-PH-GFP**

Numbers left of images denote cells positive for membrane association of TAPP1-PH-GFP/cells considered per image.

Supplemental Figure 2C. MDCK cells co-expressing RFP-PTEN(A126G, TI167/168ET) together with TAPP1-PH-GFP (page 1)

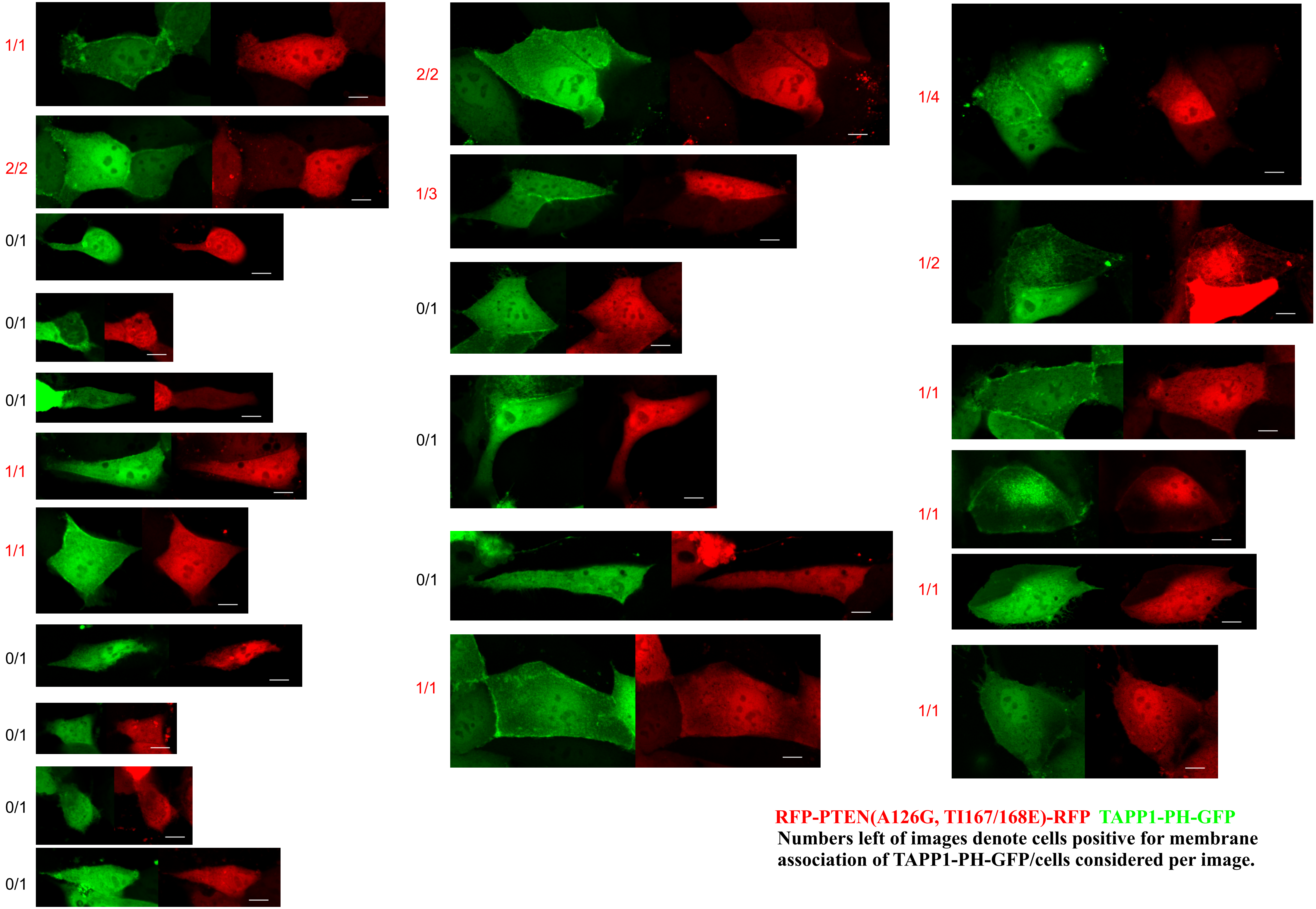

RFP-PTEN(A126G, TI167/168E)-RFP TAPP1-PH-GFP
Numbers left of images denote cells positive for membrane association of TAPP1-PH-GFP/cells considered per image.

Supplemental Figure 2C. MDCK cells co-expressing RFP-PTEN(A126G, TI167/168ET) together with TAPP1-PH-GFP (page 2)

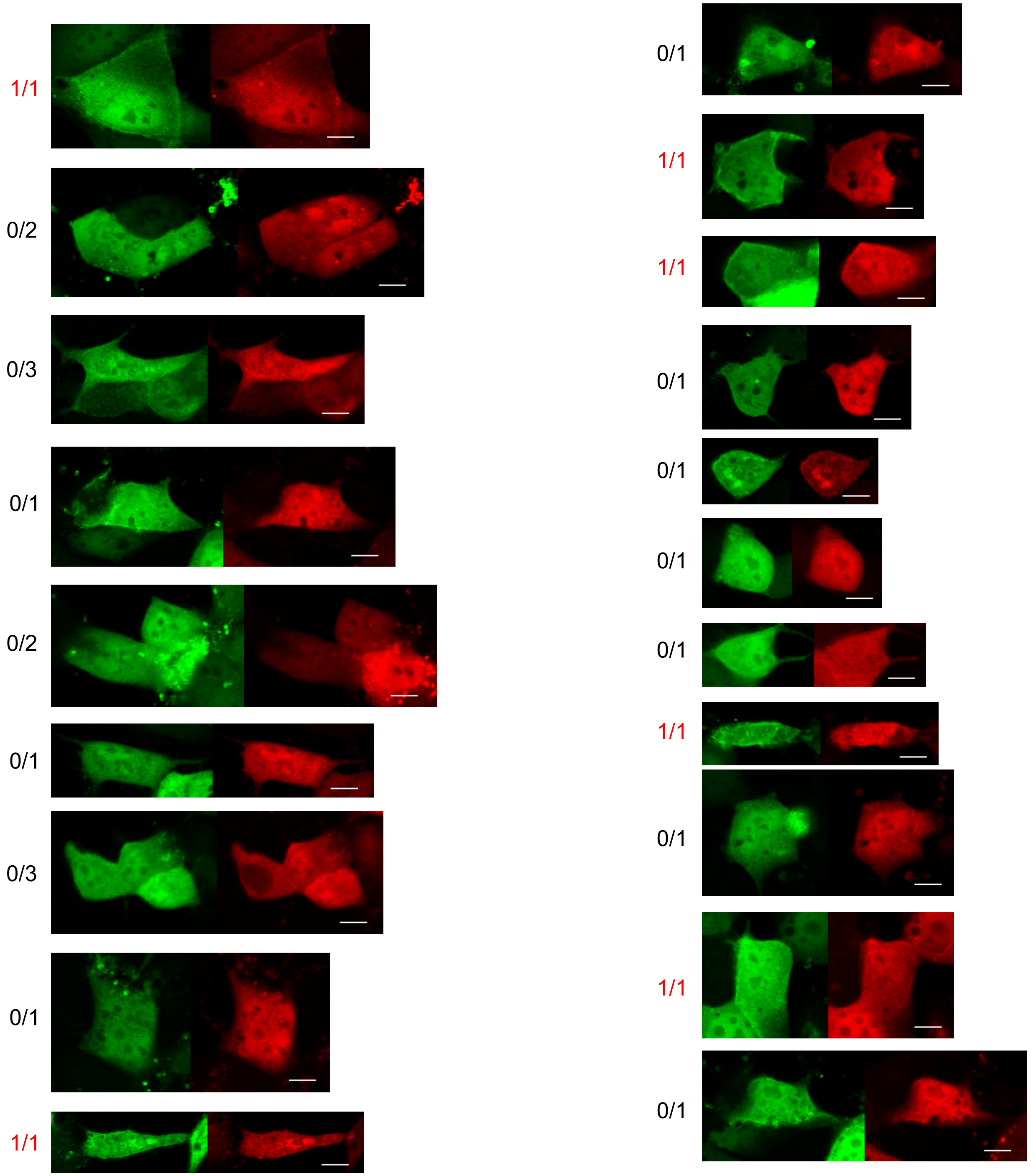

Supplemental Figure 2. Subcellular localization of TAPP1-PH-GFP in MDCK cells co-expressing soluble RFP-PTEN (wild type and mutants).

The PI(3,4)P_2-reporter TAPP1-PH-GFP was co-expressed in MDCK cells with N-terminally RFP-tagged **(A)** wild type PTEN, **(B)** PTEN(C124S) and **(C)** PTEN(A126G, TI167/168ET). Subcellular localization of the reporter was analyzed by confocal microscopy and image processing in ImageJ. This figure shows all cells analyzed in these experiments (all scale bars represent 10 µm). Quantification was done by blinded counting of cells with evident membrane association of TAPP1-PH-GFP (numbers left of single images indicate number of cells counted as positive for membrane association of TAPP1-PH-GFP/from all cells considered per image). Summary of results: PTEN(wild type): 0 of 42 cells in total (0.0%); PTEN(C124S): 1 of 36 cells in total (2.8%); PTEN(A126G, TI167/168ET): 21 of 57 cells in total (36.8%).

RFP-PTEN(A126G, TI167/168E)-RFP TAPP1-PH-GFP

Numbers left of images denote cells positive for membrane association of TAPP1-PH-GFP/cells considered per image.

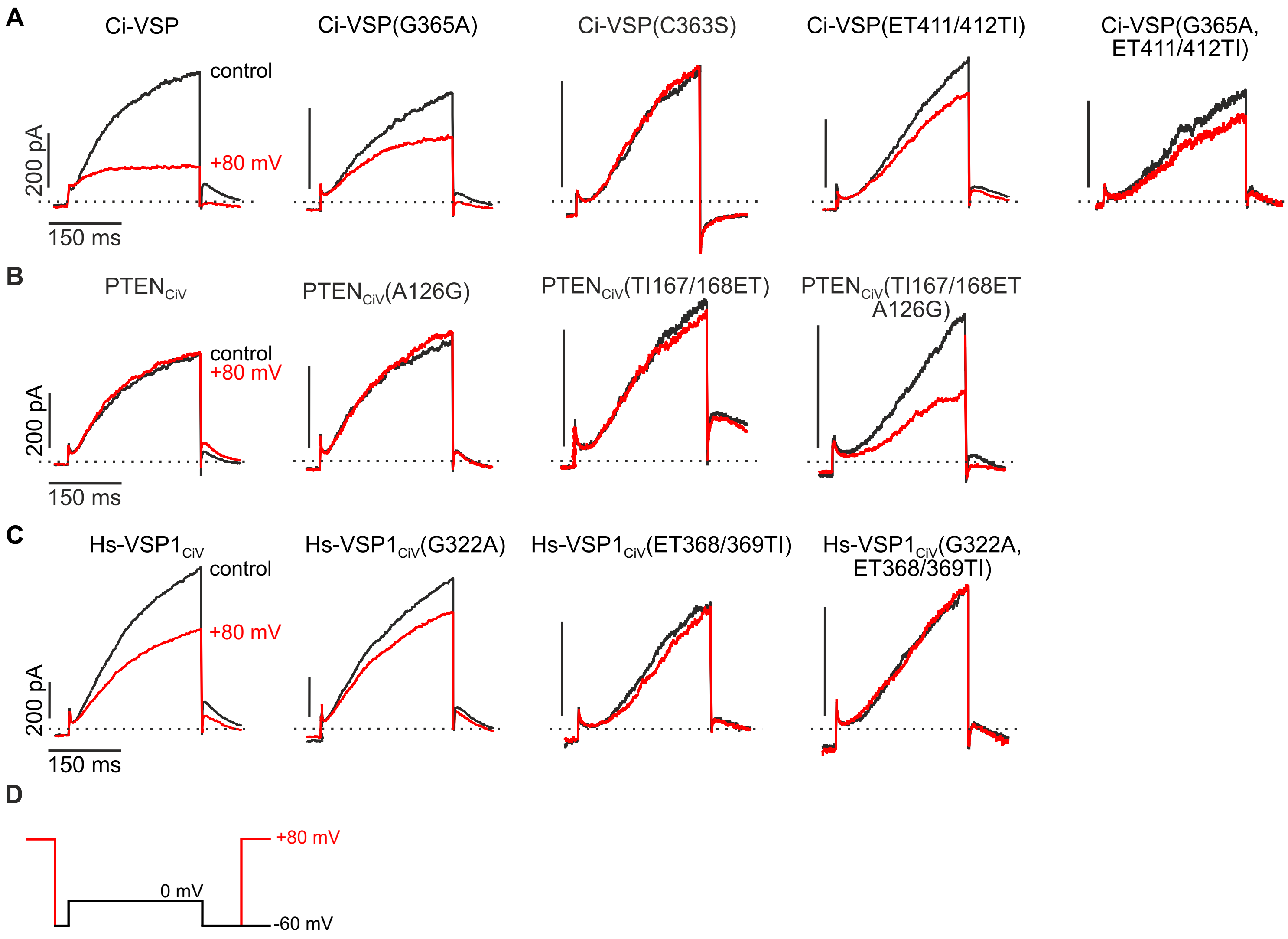

Supplemental Figure 3. Representative recordings of KCNQ2 (K_v7.2) currents in cells coexpressing VSPs. (**A**) KCNQ2 co-expressed with Ci-VSP wild type and mutants as indicated. Presented are KCNQ2 currents before (*black*) and at the end of Ci-VSP activation (*red*) (30 s at +80 mV). (**B**) Whole-cell currents in cells expressing KCNQ2 channels together with $PTEN_{CiV}$ wild type and mutants (*black* and *red* trace represent control amplitudes and at the end of $PTEN_{CiV}$ activation, respectively). (**C**) KCNQ2 currents before (*black*) and after (*red*) voltage-dependent activation of Hs-$VSP1_{CiV}$ wild type and mutants. (**D**) Voltage protocol as employed for the recordings presented in (*A-C*). CHO cells were clamped at -60 mV and KCNQ2 currents were elicited every 5 s by a step depolarization to 0 mV. Under control conditions, the holding potential between the steps was constantly kept at -60 mV. To activate VSPs, the holding potential was changed to +80 mV in between the steps (indicated in *red*). Dashed line in panels A-C represents zero current.

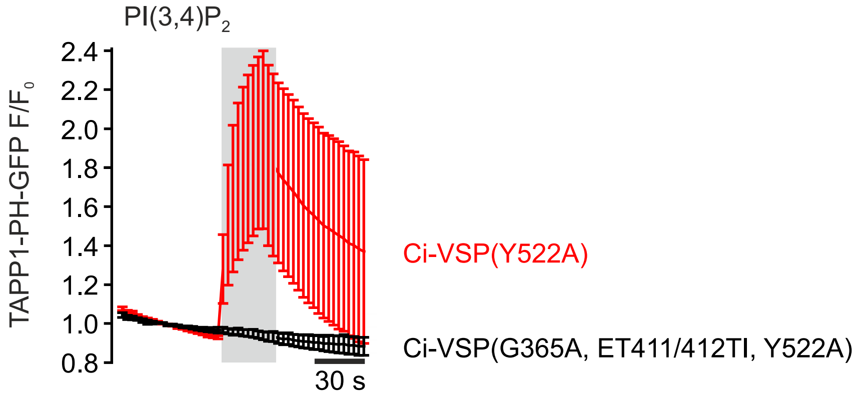

Supplemental Figure 4. Substrate specificity of Ci-VSP Y522 mutants.

Time course of the TIRF signal of TAPP1-PH-GFP co-expressed with Ci-VSP(Y522A) (*red trace,* n=5) or Ci-VSP(G365A, ET411/412TI, Y522A) (*black, n=5*). Phosphatases were activated by step depolarization to +80 mV for 30 s from a holding potential at -60 mV.
